# Dietary Nutrient Adequacy in Aeronautical Military Personnel in Spain: Strengths and Weaknesses

**DOI:** 10.3390/nu17010092

**Published:** 2024-12-29

**Authors:** Alejandra Carretero-Krug, Ana Montero-Bravo, Natalia Úbeda

**Affiliations:** 1 Grupo USP-CEU de Excelencia en Investigación “Nutrición para la Vida” (Nutrition for Life), Ref: E02/0720, Departamento de Ciencias Farmacéuticas y de la Salud, Facultad de Farmacia, Universidad San Pablo-CEU, CEU Universities, Urbanización Montepríncipe, 28660 Boadilla del Monte, Spain; amontero.fcex@ceu.es; 2 Instituto Universitario CEU Alimentación y Sociedad (IAS), Facultad de Farmacia, Universidad San Pablo-CEU, CEU Universities, Urbanización Montepríncipe, 28660 Boadilla del Monte, Spain; 3 Grupo de Investigación Consolidado “Alimentación y Nutrición en la Promoción de la Salud” (CEU-NutriFOOD), Departamento de Ciencias Farmacéuticas y de la Salud, Facultad de Farmacia, Universidad San Pablo-CEU, CEU Universities, Urbanización Montepríncipe, 28660 Boadilla del Monte, Spain; nubeda@ceu.es

**Keywords:** aeronautical military personnel, macronutrients assessment, micronutrients assessment, dietary intake, Spain

## Abstract

Background/Objectives: Aeronautical military personnel operate under intense physical and mental stress, requiring high psychophysical aptitude. Adequate nutrition is essential to sustain operational readiness and mitigate the risk of chronic diseases and other health issues. This study aims to evaluate the nutritional status of aeronautical military personnel group in Spain through dietary parameters. Methods: A total of 390 male aeronautical military personnel, who attended the *Centro de Instrucción de Medicina Aeroespacial* (Madrid, Spain), were evaluated. Energy and nutrient intakes were estimated by three non-consecutive 24-h dietary recalls (DIAL^®^ program). Results: The median energy intake was 2134 kcal/day, with significant variations across professional groups, highest in parachutists (2347 kcal/day). Protein intake was 18.2% TE, while carbohydrate intake was 37.6% TE, below standards, with 83.8% of participants not meeting the EFSA guidelines. However, a high intake of added sugars was observed (10% TE). Fat intake (38.3% TE) and saturated fats (11.7% TE) exceeded recommendations. Micronutrient deficiencies were notable for vitamin D (98% below recommendations), folate (56.4%), vitamin C (40.2%), calcium (50.3%), iodine (76.6%), and zinc (59%). Elevated cholesterol intake (352 mg/day) and inadequate fiber intake (18.5 g/day) were also observed. Conclusions: This study highlights imbalanced dietary patterns among Spanish aeronautical military personnel, with high protein, fat, cholesterol and added sugar consumption, low carbohydrate, and inadequate intake of critical micronutrients and fiber. These findings emphasize the need for targeted dietary interventions, nutritional monitoring protocols, and specific guidelines to enhance health and performance in this specialized group.

## 1. Introduction

Aeronautical military population work under severe conditions of stress, hypoxia, spatial disorientation, and physical effort. Due to these special characteristics, they have a higher risk of chronic diseases (respiratory, cardiovascular, diseases related to mental health and cancer) [1,2]. In addition, they frequently have food and sleep disorders, excessive weight, skeletal muscle pain, decreased cognitive performance, and neurological symptoms (dizziness and headaches) [3,4,5]. Owing to the complexity of the missions of the air force, high levels of psychophysical aptitude are required. Pilots (whether airplane or helicopter) who perform combat and transportation tasks are faced with several stressors, such as security threats, increased anxiety during take-off and landing, and a range of environmental factors that directly affect their performance, requiring strong physical and mental preparation [6]. In addition, parachuting in the military field is of great importance because of its use as a tactical element, and as a means of transporting troops to locations inaccessible by other methods. Skydiving is one of the most challenging activities, requiring great control of physical and mental fitness [7,8]. Both mental qualities (especially concentration) and physical preparation (strength, elasticity, and muscular endurance) are important to be able to perform demanding movements in all phases of descent and combat [9,10]. Mechanics and crew members perform repair and maintenance issues of the aircraft, even in combat situations, which will prevent accidents at different scales. Among multiple threats in aviation, tracing a technic fault in the repair and maintenance could be extremely difficult, which makes the physical and mental preparation of this staff indispensable [11].

One of the aims of military training is to prepare soldiers to accomplish demanding military missions while enduring physical and mental stress. During these military training courses, it is important to consistently maintain high levels of alertness and readiness, both from a physical and psychological perspective. Adequate performance and optimal recovery are important factors in this respect. Both factors are influenced not only by the amount of sleep and rest but by an adequate nutritional and hydration status [12,13,14]. Due to different levels of psychophysical aptitude, according to their occupation and work environment, different nutritional requirements should be achieved [15,16]. Specific military dietary references have been developed in different countries [17,18,19] but not in Spain, a traditional Mediterranean country in terms of dietary habits, lifestyle and climate conditions. The implication of having poor nutrition includes increased risk of ill health, increased associated medical care costs, a reduced number of military personnel on duty due to absenteeism, reduced operational readiness, and decreased retention of personnel [20,21]. Furthermore, long-term effects of macro- and micro-nutrient imbalances have been related to obesity, hypertension, coronary heart disease, diabetes, osteoporosis and kidney failure [22]. At the same time, these nutrient inadequacies may depress immune function, prolong recovery from illness and injury, and compromise physical and cognitive performance [23,24].

At present, there is a limited amount of research on this unique and potentially vulnerable group. The purpose of the present study is to assess the nutritional status of the aeronautical military personnel serving in the Spanish Air Force through dietary parameters.

## 2. Materials and Methods

Volunteers from all over Spain were recruited at the official *Centro de Instrucción de Medicina Aeroespacial* (CIMA, Ministry of Defense, Madrid, Spain), where aeronautical military personnel attend their annual medical check-ups.

Ethical approval was granted by the Clinical Research Ethics Committee of the CEU San Pablo University (Madrid). The corresponding ethical code was 119/16/08, and this study was performed in accordance with the ethical standards laid down in the 1964 Declaration of Helsinki and its later amendments. All the aeronautical personnel who participated in this study were voluntary and their written informed consent was obtained prior to this study. All personal data were confidential and only investigators assigned to the project had access to them.

### 2.1. Recruitment

The military aeronautical personnel attended to the CIMA for a 2- to 3-day medical check-up. Recruitment started the first day of the medical check-up and included a volunteer briefing with infographics of the procedure of this study. Volunteers that showed interest in participating in this study received the written protocol, as well as verbal answers to any questions. The inclusion criteria were as follows: all individuals who were mentally and physically healthy (which is determined through various medical consultations, included in the periodic medical check-up). Exclusion criteria were people suffering from a disease or any special health conditions that limited their flight license, regulated by Spanish law.

The initial sample included 426 subjects, 415 adult males and 11 adult women. The women were excluded due to the low sample size of this group. Of the 415 men, the participation rate was 94%. Six percent of the total subjects either did not give their consent to participate in this study or were not selected due to not meeting the inclusion criteria The final sample included 390 men (22–57 years old).

### 2.2. Study Protocol

During the first day, participants completed the self-reported questionnaires during the morning under the supervision of trained dietitian personnel.

1. Lifestyle questionnaire. Data regarding personal data, employment history and health record were obtained by a questionnaire specially designed for this study.

2. Interview/dietary intake. Dietary intake was assessed with three non-consecutive 24 h recalls (two working days, and one rest day) following the recommendations of the EU Menu Project (European Food Safety Authority (EFSA)) [25]. The first 24 h recall was assessed at the CIMA face-to-face and the other two by telephone (with a one-month period between each call). Detailed information was requested on the dietary intake of the participants, indicating the ingredients and methods of preparation in the case of culinary recipes. Energy and nutrients intakes were calculated using the DIAL^®^ software program (version 3.15). To assess macronutrient, fiber, and cholesterol adequacy, we used the following recommendations (RE): the dietary reference values from the EFSA [26], except if a recommendation has not been established, in that case we used the Spanish dietary references from Spanish Guidelines and Goals (*Sociedad Española de Nutrición Comunitaria* (SENC)) [27,28], or the dietary recommendations from the World Health Organization (WHO) [29,30]. In addition, the percentage of subjects below/above the established reference ranges was estimated.

To assess micronutrient adequacy, we used the dietary reference values (DRV) from the EFSA [26]. We used the population reference intakes (PRI), and when there was no PRI, we employed as reference the adequate intake (AI). For micronutrients, the percentage of the sample that was below/above the recommendations was estimated.

### 2.3. Statistics

Variables were tested for normality using the Kolmogorov–Smirnov test. In light of the results, data are presented as median and interquartile range (p25–p75). Differences between variables were assessed using the Kruskal–Wallis and the Mann–Whitney U test and considered significant at *p* ≤ 0.05. All statistical analyses were performed using SPSS 24.0 Software (IBM Corp., Armonk, NY, USA).

## 3. Results

A total of 390 adult males were finally included in this study, ranging between 22 and 57 years old. The sample included 81 airplane pilots (AP), 78 helicopter pilots (HP), 89 parachutists (P), 57 mechanics (M), and 85 crew members (CM).

### 3.1. Intake of Energy, Macronutrients, Dietary Cholesterol and Fiber

The values of energy and macronutrient intake (the percent of total energy intake (% TE)) and the proportions of the population that falls below and above the recommendations (RE) or meet the RE are presented in Table 1. The median energy intake was 2134 kcal/day in the total sample, with significant differences observed between the professional categories, with higher values in parachutists (2347.8 kcal/day).

Median values of protein (% TE) were 18.2 in the total sample, showing very similar values in the different professional categories. Furthermore, median values of protein (g/day) were 95.5 in the total sample, and by professional category, significantly higher intakes were observed in the group of helicopter pilots and parachutists (103 and 104.5 g/day, respectively). Taking into account the EFSA recommendation (0.83 g of protein/kg of body weight) [26], only 10.5% of the entire sample failed to achieve it.

Median values of carbohydrates were 38.4% TE for the total sample. Also, similar values were observed according to professional category, with no significant differences between groups. It should be noted that, both in the total sample and in the different occupational groups, more than 78% of the subjects had a carbohydrate intake below the EFSA recommendation (45–60% TE) [26]. However, concerning the intake of total sugars (% TE), a high consumption was observed, both in the total sample (15.5% TE) and in all professional categories. When the intake of these total sugars was broken down, it was observed that added sugars were 10.1% TE in the total sample, with very similar values in all professional categories. Taking into account WHO recommendations (<10% TE), more than 50% of the total sample had intakes above the reference value and, according to employment categories, higher levels were observed in crew members (62%).

Total fat accounted for 38.3% of total energy intake in the total sample, and similar values were observed in the different professional groups. It is important to highlight that 69% of the total sample had an intake above the EFSA recommendations (20–35% TE) [26], as well as in the different occupational categories (>62% of subjects in all groups). Fatty acids contribution to energy showed 11.7% TE from saturated fatty acids (SFA) in the total sample. In addition, between groups, significantly higher results were observed in helicopter pilots (12.5% ET). It is interesting to remark that more than 92% of the total sample, and of the subjects in the different job categories, had an intake of SFA above the SENC recommendations (7–8% TE) [27]. The percentage of TE from monounsaturated fatty acids (MUFA) was 15.7 in the total sample, and similar values were observed in the different professional categories. Considering the SENC limit (>20% TE) [27], a high percentage of the total sample and of the different occupational classifications (>82%) had an intake of MFA below recommendations. The intake of polyunsaturated fatty acids (PUFA) was 6.8% TE in the total sample, and similar values were observed in the different professional categories. Of the total sample, 66% had an intake above the Spanish recommendation (5% TE) [27], with a higher percentage of inadequacy to the recommendations in the group of airplane pilots (79%). Furthermore, the intake of trans fatty acids (TFA) in the total sample represented merely 0.3% TE, and similar results were observed in the different employment categories. These values indicate that a high percentage of the total population and of the different professional categories (~99%) had a TFA intake below the SENC nutritional goal (<1% TE) [27]. Dietary cholesterol intake (Table 1) in the total sample was 352 mg/day, and similar results were observed for the different job groups. Moreover, 65% of the total sample was above the Spanish nutritional goal establish by the SENC (≤300 mg/day) [27], and similar results were observed by occupational groups, highlighting even higher values in parachutist (71%).

Fiber intake was 18.5 g in the total sample, similar in all professional categories. Of the total sample and the different professional categories, 79% were below the European recommendations for fiber (25 g/day, EFSA) [26].

Table 1 also shows the intake of fatty acids omega-3 and omega-6. The intake of fatty acids omega-3 in the total sample was 0.5% TE, and similar results were observed by professional category [27]. It can be observed that 82% of the total sample and of the different occupational categories had omega-3 intakes below the nutritional goal of the SENC (1–2% TE) [27]. Specifically, the intake of eicosapentaenoic acid (EPA) together with the intake of docosahexaenoic acid (DHA) was 275.1 mg/day in the total sample, similar in all groups. Around 39% of the total sample and different employments showed intakes below the European recommendations (250 mg/day, EFSA) [26].

### 3.2. Intake of Micronutrients and Prevalence of Adequation

The intake of vitamins and minerals are presented in Table 2 and Table 3, as well as the percentage of the total sample and groups that complies with the DRV or below the DRV. Regarding vitamin intake, Table 2 shows significant differences between professional categories for all vitamins, except for folate, vitamin B12, vitamin C, vitamin E, and vitamin K. It should be noted that the intake of fat-soluble vitamins was generally adequate, except for vitamin D [26]. There was observed a very low intake of vitamin D since > 97% of the total sample and the different job groups did not meet the EFSA recommendations (15 µg/day) [26]. However, it is important to highlight that more than one third of the total population did not meet the EFSA recommendations for vitamin A (750 μg ER/day) and vitamin E (13 mg α-TE/day) [26]. By professional category, a higher percentage of inadequacy was observed in the crew members group for both vitamin A (41%) and vitamin E (46%). On the other hand, the intake of water-soluble vitamins was generally adequate, except for folate and vitamin C. It should be noted that half of the subjects in the total sample and the different employment categories did not reach the EFSA recommended folate intake (330 μg/day) [26], and 40% of the total sample had vitamin C intakes lower than the EFSA’s recommendations (110 mg/day) [26]. By professional category, the airplane pilots stood out with a lower prevalence of inadequacy (28%).

Concerning mineral intake (Table 3), significant differences were observed among professional categories for iron, zinc, phosphorus, and selenium. Overall, mineral intake was only adequate for iron, phosphorus, and selenium, indicating that over 90% of the total sample and professional categories reached the recommendations of the EFSA. A high prevalence of inadequacy intake, more than a half, was noted for calcium, iodine, and zinc, and around 40% for magnesium and potassium. Although a similar prevalence of inadequacy was observed by groups, the helicopter pilots highlighted with a lower prevalence of inadequacy for calcium (37%). Furthermore, the airplane pilots showed a higher prevalence of inadequacy for zinc (72.4%).

The total sodium intake in the sample was 2084.2 mg/day (Table 3). It should be noted that this study has not taken into account the salt added to the foods. More than half of the total sample consumed amounts exceeding the EFSA’s recommended 2000 mg/day [26], with the parachutist group notably standing out among professional categories, as 70% of its members surpassed this recommendation. 

## 4. Discussion

This study provides the most complete and updated data of energy and nutrient intake in aeronautical military personnel in Spain. It includes a classification according to professional category, which is of great value and utility for detecting possible deficiencies and/or nutritional imbalances related to their profession. Dietary needs in military personnel differ from civilian requirements due to varied physical and mental workloads associated with their occupation and work environment [16,31].

The energy intake of this study group (2134 kcal/day) is comparable to the Spanish civilian population (1966 kcal/day, ANIBES study) [32], and to the administrative military population of Europe (2389 kcal/day) [33]. However, it is notably lower than the energy intake observed in aeronautical military personnel from other countries (US, China, and Israel) (2500–3200 kcal/day) [34,35,36,37], and in other military units (US, Belgium, and Czech Republic) (2600–4000 kcal/day) [12,31,38,39]. Compared to the EFSA recommendations (2600 kcal/day, moderately active lifestyle), the total intake of the sample and different professional categories did not meet the recommendations. This result was anticipated in previous research [40], which shows that military personnel often consume insufficient energy during periods of military training, regardless of whether an adequate amount is provided. The parachutist group showed a slight but significant increase in caloric intake (2347 kcal/day), likely reflecting a greater physical demand associated to professional categories.

One of the main dietary quality indices used is the macronutrients contribution to total energy. In this study, protein intake contributed 18.2% of TE, with similar data in the different jobs, higher than in the Spanish civilian population [32,41,42], and international military population (14–16.7% TE) [12,31,33,34,36,37,38,43,44,45], but similar to US Army Rangers [46].

Lipid intake was 38.3% TE in this study and was similar between groups, as well as was also reported in the ANIBES study [32], and in a study conducted on US Army Rangers [46], surpassing the EFSA [26] and the SENC [27] recommendations. However, a higher intake was observed in Chinese military air personnel (46% TE) [34], while lower values (33–36% TE) were observed in international military population [12,31,33,35,36,37,38,43,44,45]. The intake of SFA in the total sample was 11.7% TE, with slightly higher values in helicopter pilots (12.5% TE), again exceeding the SENC recommendations [27]. This aligns with the ANIBES study [32], the US military population [31,35,37], and the Finnish military population [38]. Higher SFA values were observed in Belgian military personnel (13–14%) [12,33], while lower values were observed in Israeli Air Force pilots (9.5%) [36]. In this study, MUFA contributed 15.7% to total energy intake (with similar data across all professional categories), similar to the Spanish civilian population (16.6% TE, ANIBES study) [32], but lower than the SENC [27] and the FAO/WHO [29] recommendations. However, this study showed higher values than other studies conducted on military personnel from different countries (11.4–12.3% TE) [31,33,36,37,38], likely due to the use of olive oil in the diet, influenced by the traditional Mediterranean diet. On the other hand, PUFA contributed 6.8% to the TE of the sample in this study, being similar in occupational categories, and exceeding the SENC recommendations [27]. Similar results were observed in the ANIBES study [32], as well as in other studies conducted on military personnel from the US [31,37] and Belgium [33]; higher data were observed in Israeli Air Force pilots (10.6% TE) [36]. However, levels lower than the results of this study and within European recommendations were observed in the Finnish military population [38]. The combined intake of EPA and DHA in this study was 275 mg/day in the total sample, with very similar intake across the professional categories, accomplishing the recommendations of the EFSA (250 mg/day) [26]. Moreover, it has been observed that an ω-6/ω-3 ratio of 2-5/1 [47] may be beneficial for health. However, neither the EFSA nor the SENC made any recommendations [26,27]. In this study, the ω-6/ω-3 ratio was 10/1 in the total sample, with similar values across professional categories, similar values to those observed in a study conducted in Spain by the Ministry of Agriculture, Fisheries, and Food, which demonstrated that, despite the Spanish population consuming omega-3 fatty acids in amounts close to the recommendations (1.5 g/day), the ω-6/ω-3 ratio was very high (16:1) [42]. Ratios of 10/1 or even 20–25/1 promote the pathogenesis of many diseases, including cardiovascular diseases, cancer, and inflammatory and autoimmune diseases [48].

The cholesterol intake was 352 mg/day, similar in professional categories, as well as in a study conducted in the British Navy [45]. In all cases, levels were higher than the SENC recommendations (<300 mg/day) [27]. On the other hand, higher cholesterol intakes were observed in Israeli Air Force pilots [36] and US special forces [37], while lower intakes, within the recommendations, were observed in military population from the US and Belgium [31,33].

In this study, a low percentage of energy from carbohydrates was observed (37.6%), well below the EFSA (45–60% TE) [26] and the SENC (50–55% TE) [27] recommendations, which could reduce the availability of the quick energy needed in situations of high physical demand or prolonged mental stress. Similar results were observed in Chinese military aviation personnel [34]. However, higher values, although mostly below the recommendations, were observed in the Spanish civil population (41.1% TE) [32], as well as in other studies (42–51% TE) conducted in military populations from different countries [12,31,33,35,36,37,38,43,44,45,46]. Furthermore, approximately 10% of total energy intake in the total sample and across the professional categories were provided by extrinsic/added sugars. These values are at the limit of the 10% TE, and they were double the recommendation of 5% TE, which would offer additional health benefits, according to the WHO recommendations [30]. Excessive sugar intake is currently recognized as a risk factor for obesity, type 2 diabetes, cardiovascular diseases, and cancer [49]; it also stands out as a factor that disrupts the gut microbiota and the immune system [50]. To date, in military personnel, only the association between the consumption of sugary beverages and a lower quality of life has been studied [51].

Adequate fiber intake has health benefits, including maintaining body weight, reducing the risk of cardiovascular diseases, type 2 diabetes, and certain types of cancer [52]. However, the fiber intake in the present study showed values well below the EFSA (>25 g/day) [26] and the SENC (>35 g/day) [27] recommendations for adult men. Similar fiber intakes (13–23 g/day) were observed in military personnel [31,33,36,37,43,45] and in the Spanish civilian population (13.1 g/day) [53].

The deviation from ideal macronutrient ratios suggests a potential shift away from balanced diets, which may be influenced by the convenience of protein and fat-dense foods and limited access to complex carbohydrates during deployment or training.

Recent research on micronutrient intake highlights widespread deficiencies in vitamins and minerals due to poor diet quality [54]. In Spain (Ministry of Agriculture, Fisheries, and Food) zinc, magnesium, folate, and vitamin B6 intakes often fall below the recommended levels (20–40 years old) [55]. The ANIBES study [54] also noted insufficient consumption of magnesium, zinc, calcium, folate, vitamin A, vitamin E, and vitamin D. Our study similarly finds that deficient intake in vitamins D, C, A, E, folate, and minerals like calcium, iodine, zinc, magnesium, and potassium, which can impact both physiological and psychological performance. Starting a military operation with an inadequate micronutrient status can put individuals at risk, leading to impairments in physiological and psychological function and performance, especially when operations/missions occur repeatedly without adequate replenishment [56].

Vitamin D deficiency is common globally; with low intakes observed in our study (2.5 μg/day), very similar to those described in the US Navy (4.5 μg/day) [43] and in recruits of the British Navy (2 μg/day) [45]. For this reason, vitamin D supplementation is increasing in the military population [57], as it has been shown to improve bone health [58] and maintain immunity [59]. Folate was also below the EFSA recommendations (330 μg/day) [26], aligning with the British Navy data [45], while higher results, closer to the EFSA recommendations, were observed in military personnel from the US [31,35,37,43,60] and Israel [36]. Adequate folate and vitamin B12 intake are crucial for preventing cardiovascular and certain neurological diseases [61,62]. Vitamin B12 also helps protect against noise-induced hearing loss [63], as shown in studies on military personnel and pilots, which found a link between hearing loss and levels of folate and homocysteine [64]. Our study found vitamin B12 intake to be adequate as well as in international military population [31,36,43,45] and the Spanish civilian population [65]. Other B-group vitamins, such as thiamine, riboflavin, niacin, and vitamin B6 showed adherence to the EFSA recommendations [26], similar to findings for military personnel from the US [35,37,43], the UK [45], China [34], and Belgium [33]. In this study, vitamin E (12.6 mg/day) was very close to the EFSA recommendations (13 mg/day) [26], which is near to studies conducted in military populations from the US [31] and Israel [36]. By contrast, lower vitamin E intake (<10 mg/day) was observed in other studies conducted in military personnel from the US [35,37,43], and in Spanish men from the ANIBES study [65]. Vitamin C intake was below the EFSA recommendation (110 mg/day) [26], but above the Spanish recommendation (60 mg/day) [66]. Similar results were found in Spanish civilian men [65] and in military personnel from the UK [45], Belgium [33], and the US [35]. However, higher intakes were reported in military personnel from the US [43], Israel [36], and China [34]. And vitamin A intake was below the EFSA recommendation (750 μg RE/day) [26], only airplane pilots met the recommendation. Higher intakes (800–1400 μg RE/day) were found in military studies from the US [31,37,43], the UK [45], China [34], and Israel [36], while lower intakes (<700 μg RE/day) were observed in a US airborne assault division [35] and Spanish men (ANIBES study) [65].

Regarding minerals, the intake of calcium was similar to data observed in a group of Spanish men [67], and in several studies conducted in military populations [33,34,35,36,45]; in all cases it was lower than the EFSA recommendations (950–1000 mg/day) [26]. However, higher values (within the EFSA reference ranges) were observed in different studies in military personnel from the US [31,37,43]. Gaffney-Stomberg et al. (2014) [58] conducted a randomized, double-blind, placebo-controlled trial in military personnel, where one of the groups received calcium and vitamin D supplementation during their basic combat training. This study observed that calcium and vitamin D protected bone health during training, thus reducing the risk of stress fractures. Besides that, in this study, the zinc intake was also below the EFSA recommendation (14 mg/day) [26], similar to results observed in the ANIBES study [65] and in studies conducted in military populations [35,36,43,45]. However, higher results within European reference values were observed in studies conducted in US special forces population (16.3 mg/day) [37], and Chinese military air personnel (18 mg/day) [34]. Iodine intake was below the EFSA recommendation (150 μg/day) [26], consistent with findings from other military studies [34,35,45], reflecting a common mild deficiency in Europe [68]. Magnesium intake was below the EFSA recommendation of 350 mg/day [26], a result also observed in Spanish civilian males [67] and various military populations [36,37,43,45], except for Chinese military [34] air personnel who met the recommendations. Magnesium is a crucial mineral for over 300 enzymatic reactions, muscle contraction, gland secretion, nerve transmission, and activating vitamin D [69], and protects against noise-induced hearing damage, which is significant for populations exposed to high-frequency sounds [63]. Iron intake generally met the EFSA recommendations in this study [26], similar to observations in other military studies [31,33,34,35,36,37,43]. However, lower intakes were observed in Spanish males [70] and the UK navy [45]. The results highlight that sodium intake was high (2084 mg/day), consistent with intakes across the ANIBES study [71] and other military populations [31,33,34,35,36,37,43,45]. Although it should be noted that military personnel often engage in more physical exercise, so considering athlete recommendations (American College of Sports Medicine), which suggest 300–600 mg of sodium per hour during exercise, might be relevant [72]. Even so, potassium intake was below the EFSA recommendation (3500 mg/day) [26], similar to results of other military studies [34,35,43,45]. But higher intakes were observed in some US military bases [31,37] and Belgian military administrative personnel [33]. Elevated sodium intake and inadequate potassium intake are linked to health risks, like high blood pressure, cardiovascular diseases, and kidney diseases [73].

The prevalence of these deficiencies indicates that current dietary intake may not be sufficient to meet the physiological demands placed on Spanish military personnel. These nutrient gaps present a more pressing issue for military personnel, whose cognitive performance, recovery times, and resistance to illness are paramount to mission success.

Strengths and limitations: The present study has several strengths. First, there are limited studies among military personnel in Spain that concurrently assessed the dietary intake (macro and micronutrients). In that way, this study represents a unique exploration of dietary intake in aeronautical military personnel in Spain. Second, we have compared the results between different military groups, using the same methodology. Third, we used a validated method for dietary assessment.

The main limitation is the relatively small group of each professional category. Another point to consider is the limited number of women who were recruited. Since their representation was not substantial, we had to exclude them. This is because, although more women are entering the military, they still make up a minority in the army. In addition, the questionnaires have been supervised by trained dietitians and nutritionists but are self-reported, with the consequent potential bias (e.g., under-reporting). Furthermore, the lack of body composition and physical assessment data represents a limitation of this study.

## 5. Conclusions

This study presents, for the first time, dietary intake data of the Spanish aerospace military population, revealing that the dietary intake of this group is imbalanced in a similar manner to what has been observed in other military populations and in the civilian population in Spain. The diets feature high fat, protein, cholesterol, and simple sugar content, low carbohydrate intake, and insufficient fiber intake. In addition, an insufficient intake of vitamin D, folate, vitamin C, calcium, iodine, zinc, magnesium, and potassium deserves special attention.

These findings emphasize the need for targeted interventions in the military population to ensure adequate diet intake to support health and operational readiness. The development of long-term nutritional monitoring protocols and specific dietary guidelines for military personnel and their different occupations or special needs would result in improved nutritional and health status for this group.

## Figures and Tables

**Table 1:** Intake of energy, macronutrients, dietary cholesterol, fiber and prevalence of adequation to recommendations in the total sample and by professional category.

	RE		Total	Crew Members	Mechanics	Airplane Pilots	Helicopter Pilots	Parachutists	*p* Values
n			390	85	57	81	78	89	
Age (years)		median (IQ)	35 (30–43)	38.5 ^a^ (33.3–45)	46 ^b^ (36.3–51.8)	31 ^c^ (27–38.8)	33 ^d^ (30–40)	32 ^c, d^ (30–39)	<0.001
Energy (kcal/día)		median (IQ)	2134 (1799.5–2506.8)	2006 ^a^ (1786.4–2293.5)	2010 ^a^ (1808.8–2380.2)	2031.5 ^a^ (1720.5–2360.5)	2150 ^a^ (1811–2519.9)	2347.8 ^b^ (1960.2–2730.9)	<0.001
Protein (%TE)		median (IQ)	18.2 (15.9–20.3)	17.9 (15.8–20.1)	17.2 (15.3–19.1)	18.2 (15.9–19.7)	18.9 (16.3–21.7)	18.2 (15.5–20.5)	0.146
Protein (g/day)		median (IQ)	95.5 (80.6–113.5)	93.9 ^a^ (74–108)	92.3 ^a^ (79.9–101)	89.8 ^a^ (78.8–104.6)	103 ^b^ (86.2–114.7)	104.5 ^b^ (85–126)	<0.001
Carbohydrates (%TE)		median (IQ)	37.6 (33.3–42.3)	38.5 (33–43.8)	36.1 (32.9–41.8)	37.7 (34.4–41.4)	36.4 (31.9–39.5)	37.8 (33.5–44.2)	0.165
EFSA	45–60	<45% TE	83.8%	82.4%	83.6%	86.8%	89.6%	77.5%	0.331
		45–60% TE	16.2%	17.6%	16.4%	13.2%	10.4%	22.5%	
Sugars (%TE)		median (IQ)	1.5 (12.3–18.8)	16.6 ^a^ (13.2–19.3)	16.1 ^a^ (12.1–20.3)	15.7 ^a^ (13–19.1)	13.7 ^b^ (10.5–17.3)	15.2 ^a, b^ (11.2–19)	0.031
Added sugars (%TE)		median (IQ)	10.1 (7.7–12.9)	10.8 (8.3–14.1)	10.3 (8.5–12.1)	9.7 (6.9–12.9)	10 (6.8–12.9)	10 (8–13)	0.314
WHO	10	<10% TE	49.1%	38.2%	49.1%	57.9%	49.3%	50%	0.232
		≥10% TE	50.9%	61.8%	50.9%	42.1%	50.7%	50%	
Lipids (%TE)		median (IQ)	38.3 (34.1–42.8)	37.1 (33.7–41.6)	39.5 (33.8–43.6)	38.6 (34.6–42.4)	39.6 (35.3–43.5)	38 (33.8–41.8)	0.327
EFSA	20–35	>35% TE	68.8%	61.8%	69.1%	71.1%	76.1%	66.3%	0.453
		20–35% TE	31.2%	38.2%	30.9%	28.9%	23.9%	33.8%	
SFA (%TE)		median (IQ)	11.7 (10.2–13.7)	11.6 ^a, b^ (10–13.6)	11.7 ^a, b^ (10.4–13.9)	11.2 ^a^ (9.8–12.6)	12.5 ^b^ (10.6–14.5)	11.8 ^a^ (9.7–13.7)	0.032
SENC	7–8	<7% TE	1.4%	1.5%	1.8%	2.6%	-	1.3%	0.791
		>8% TE	94.5%	92.6%	92.7%	92.1%	98.5%	96.3%	
		7–8% TE	4%	5.9%	5.5%	5.3%	1.5%	2.5%	
MUFA (%TE)		median (IQ)	15.7 (13.8–18)	15.4 (13.3–17.2)	15.6 (14.1–18.8)	16.2 (13.8–18.3)	16.2 (14.3–17.8)	15.4 (13.4–18.3)	0.459
SENC	≥20	< 20% TE	88.7%	92.6	81.8%	92.1%	89.6%	86.3%	0.280
		≥ 20% TE	11.3%	7.4%	18.2%	7.9%	10.4%	13.8%	
PUFA (%TE)		median (IQ)	6.8 (4.9–8.2)	6.3 (4.7–8.1)	6.3 (4.8–8.4)	7.3 (5.7–8.5)	7 (5.2–7.9)	6.2 (4.9–7.7)	0.067
SENC	5	<5% TE	14.7%	20.6% _a_	16.4% _a, b_	7.9% _b_	14.9% _a, b_	15% _a, b_	0.045
		>5% TE	65.9%	57.4% _a, b_	63.6% _a, b, c_	78.9% _c_	73.1% _b, c_	56.3% _a_	
		~5% TE	19.4%	22.1% _a, b_	20% _a, b_	13.2% _b_	11.9% _b_	28.8% _a_	
TFA (%TE)		median (IQ)	0.3 (0.1–0.4)	0.3 (0.1–0.4)	0.2 (0.1–0.4)	0.3 (0.1–0.5)	0.2 (0.1–0.4)	0.3 (0.2–0.5)	0.072
SENC	<1	<1% TE	99.4%	98.5%	100%	100%	100%	98.8%	0.605
		≥1% TE	0.6%	1.5%	-	-	-	1.3%	
Omega-6 (g/day)		median (IQ)	10.5 (5.3–15.4)	8 ^a^ (1.2–14.1)	10.6 ^a^ (4.3–14.3)	9.7 ^a^ (6.5–14.6)	9.6 ^a^ (3.4–13.8)	13.7 ^b^ (9–18.9)	0.001
Omega-3 (mg/day)		median (IQ)	1100 (100–1827.4)	405 ^a^ (50.7–1707.5)	1000 ^a^ (65–1900)	987 ^a^ (235–1747)	586.2 ^a^ (56–1600)	1585.9 ^b^ (1024.2–2235.6)	<0.001
Omega-3 (%TE)		median (IQ)	0.5 (0.05–0.7)	0.2 ^a^ (0.02–0.6)	0.4 ^a^ (0.03–0.8)	0.5 ^a^ (0.1–0.8)	0.3 ^a^ (0.03–0.7)	0.6 ^b^ (0.4–0.8)	0.001
SENC	1–2%	1–2% TE	11.8%	10.6% _a, b_	17.5% _b_	13.6% _a, b_	17.9% _b_	2.2% _a_	0.012
		<1% TE	88.2%	89.4% _a, b_	82.5% _b_	86.4% _a, b_	82.1% _b_	97.8% _a_	
EPA (20:5) (mg/day)		median (IQ)	52.3 (7–153.6)	79.3 (10–155.6)	48 (3.8–130)	72.5 (11–240)	73.4 (8.5–162.9)	22.5 (6.1–76.5)	0.055
DHA (22:6) (mg/day)		median (IQ)	219 (110–401.8)	227.5 (97.5–387.5)	200 (77–300)	216 (121.5–525.5)	260 (97–500)	210 (117.1–336.8)	0.508
EPA + DHA (mg/day)		median (IQ)	275.1 (132.6–547.4)	295.7 (135.4–544.9)	232 (93.5–371)	258.5 (148.9–780)	325.3 (112.5–660)	237 (129.9–430)	0.427
EFSA	250	<250 mg/day	47.4%	39.7%	52.7%	48.7%	43.3%	52.5%	0.466
		≥250 mg/day	52.6%	60.3%	47.3%	51.3%	56.7%	47.5%	
Omega-6/Omega-3		median (IQ)	10 (6–7)	11 (4–20)	10 (6–19)	11 (7–22)	10 (6–24)	9 (5–12)	0.060
Dietary cholesterol (mg/day)		median (IQ)	352 (264.8–464)	332.7 (258.8–441)	318.8 (267.2–458.2)	322.3 (260–444.2)	378 (270.7–456)	401.4 (277.4–516.8)	0.126
SENC	300	≤300 mg/day	35.3%	38.2%	43.6%	36.8%	31.3%	28.8%	0.410
		>300 mg/day	64.7%	61.8%	56.4%	63.2%	68.7%	71.3%	
Fiber (g/day)		median (IQ)	18.5 (13.5–23.1)	17.1 (12.5–22.3)	19.4 (13.3–24.7)	17.5 (14.4–21.9)	18.4 (11.9–22.6)	19.8 (15.8–24.6)	0.198
EFSA	>25	<25 g/day	81.2%	79.4%	80%	85.5%	82.1%	78.8%	0.831
		≥25 g/day	18.8%	20.6%	20%	14.5%	17.9%	21.3%	

Results are presented as median and interquartile range (IQ). *p*-values derived through Kruskal–Wallis (*p* ≤ 0.05). ^a, b, c, d^ significant differences between groups (Mann–Whitney U test). _a, b, c_ significant differences between groups (Chi^2^). RE—recommendations. TE—Total energy. SENC—Sociedad Española de Nutrición Comunitaria. EFSA—European Food Safety Authority. WHO—World Health Organization. SFA—Saturated fatty acids. MUFA—Monounsaturated fatty acids. PUFA—Polyunsaturated fatty acids. TFA—Trans fatty acids. EPA—Eicosapentaenoic acid. DHA—Docosahexaenoic acid.

**Table 2:** Intake of vitamins and prevalence of adequation to the recommendations in the total sample and by professional category.

	PRI/AI		Total	Crew Members	Mechanics	Airplane Pilots	Helicopter Pilots	Parachutists	*p* Values
n			390	85	57	81	78	89	
Thiamine (mg/day)		median (IQ)	1.4 (1.1–1.8)	1.4 ^a, b^ (1.1–1.8)	1.3 ^a, b^ (1–1.8)	1.3 ^a^ (1.1–1.6)	1.6 ^b^ (1.2–1.8)	1.5 ^b^ (1.2–1.9)	0.008
EFSA	0.1 mg/MJ	below PRI	1.20%	-	3.6%	-	-	2.5%	0.153
		above PRI	98.8%	100%	96.4%	100%	100%	97.5%	
Riboflavin (mg/day)		median (IQ)	1.7 (1.4–2)	1.8 ^a, c^ (1.3–2.2)	1.7 ^b, c^ (1.3–1.9)	1.6 ^b^ (1.3–1.8)	1.7 ^a, c^ (1.5–2)	1.9 ^a^ (1.4–2.7)	0.004
EFSA	1.6	below PRI	15.6%	19.1%	18.2%_a_	18.4%	7.5%	15%	0.312
		above PRI	84.4%	80.9%	81.8%	81.6%	92.5%	85%	
Niacin (mg/day)		median (IQ)	40.5 (33.5–49.1)	40.2 ^a, c^ (32.8–46.9)	37.9 ^a^ (31.5–42.9)	38.6 ^a^ (32.6–44.1)	42.7 ^b, c^ (34.4–52.6)	44.1 ^b^ (35.3–57.6)	<0.001
EFSA	16	above PRI	100%	100%	100%	100%	100%	100%	-
Vitamin B6 (mg/day)		median (IQ)	2.3 (1.9–2.8)	2.3 ^a, b^ (1.8–2.7)	2 ^a^ (1.9–2.6)	2.1 ^a^ (1.8–2.5)	2.5 ^b^ (1.9–3)	2.6 ^b^ (1.9–3.4)	0.002
EFSA	1.7	below PRI	5.5%	11.8%	3.6%	5.3%	3.0%	3.8%	0.146
		above PRI	94.5%	88.2%	96.4%	94.7%	97%	96.3%	
Folates (μg/day)		median (IQ)	252 (198.6–317.2)	242 (193–284.5)	246.2 (178.5–330.8)	247.2 (207.8–319.4)	258.4 (196–322)	263 (214–339.8)	0.345
EFSA	330	below PRI	56.4%	63.2%	56.4%	59.2%	53.7%	50%	0.548
		above PRI	43.6%	36.8%	43.6%	40.8%	46.3%	50%	
Vitamin B12 (μg/day)		median (IQ)	6.2 (4.4–8.2)	6.4 (4.7–8.5)	5.4 (3.6–7.9)	6 (4.8–7.7)	6.2 (4.3–7.6)	6.6 (4.7–10.1)	0.165
EFSA	4	below PRI	10.4%	8.8% _a, b, c, d_	20% _c, d_	11.8% _b, d_	3% _a_	10% _a, b, c, d_	0.045
		above PRI	89.6%	91.2% _a, b, c, d_	80% _c, d_	88.2% _b, d_	97% _a_	90% _a, b, c, d_	
Vitamin C (mg/day)		median (IQ)	99.7 (66.2–140)	104 (69–144.2)	94.7 (55.3–162.1)	105.8 (76–139.8)	92.9 (52.1–126)	94.2 (72.8–144.8)	0.347
EFSA	110	below PRI	40.2%	39.7%	45.5%	27.6%	46.3%	43.8%	0.132
		above PRI	59.8%	60.3%	54.5%	72.4%	53.7%	56.3	
Vitamin A (μg RE/day)		median (IQ)	721.2 (554–930.5)	655.6 ^a^ (497.8–849.7)	692.9 ^a, b^ (540–908)	811.8 ^b^ (617.6–1020.4)	689.9 ^a, c^ (518–890)	734 ^b, c^ (567.5–1000)	0.021
EFSA	750	below PRI	30.9%	41.2%	29.1%	19.7%	34.3%	31.3%	0.083
		above PRI	69.1%	58.8%	70.9%	80.3%	65.7%	68.8%	
Vitamin D (μg/day)		median (IQ)	2.5 (1.4–4.3)	2.7 ^a^ (1.2–4.4)	1.6 ^b^ (1–2.9)	2.8 ^a^ (1.7–5)	2.4 ^a^ (1.3–4.5)	2.6 ^a^ (1.7–4)	0.010
EFSA	15	below AI	98%	97.1%	98.2%	97.4%	98.5%	98.8%	0.941
		above AI	2%	2.9%	1.8%	2.6%	1.5%	1.3%	
Vitamin E (mg α-TE/day)		median (IQ)	12.6 (8.6–16.4)	11.1 (7.9–15.5)	11.2 (8.4–15.9)	13.7 (9.4–17.8)	11.8 (8.8–16.4)	12.8 (9.2–17.1)	0.188
EFSA	13	below AI	37%	45.6%	43.6%	27.6%	37.3%	33.8%	0.170
		above AI	63%	54.4%	56.4%	72.4%	62.7%	66.3%	
Vitamin K (μg/day)		median (IQ)	102.9 (71.7–150.3)	101.5 (73–133.8)	106.2 (75.2–149.8)	101.8 (71.9–144.5)	117 (70.7–172.2)	99.6 (70–149)	0.644
EFSA	70	below AI	13%	11.8%	12.7%	14.5%	10.4%	15%	0.923
		above AI	87%	88.2%	87.3%	85.5%	89.6%	85%	

Results are expressed as the median (IQ). *p*-values derived through Kruskal–Wallis tests (*p* ≤ 0.05). ^a, b, c^ significant differences between groups (Mann–Whitney U test). _a, b, c, d_ significant differences between groups (Chi^2^). PRI—Population reference intake. AI—Adequate intake. EFSA—European Food Safety Authority.

**Table 3:** Intake of minerals and prevalence of adequation to the recommendations in the total sample and by professional category.

	PRI/AI		Total	Crew Members	Mechanics	Airplane Pilots	Helicopter Pilots	Parachutists	*p* Values
n			390	85	57	81	78	89	
Calcium (mg/day)		median (IQ)	758 (605–950)	746 (576–948.8)	719 (620–902)	697.4 (528.7–913.8)	840 (675–962)	843.3 (605.2–1060.5)	0.065
EFSA	950–1000 *	below PRI	50.3%	51.5% _a, b, c_	63.6% _c_	57.9% _b, c_	37.3% _a_	43.8% _a, b_	0.021
		above PRI	49.7%	48.5% _a, b, c_	36.4% _c_	42.1% _b, c_	62.7% _a_	56.3% _a, b_	
Iron (mg/day)		median (IQ)	13.7 (11.3–16.4)	13.2 ^a^ (10.1–16.2)	12.9 ^a^ (10.9–15.3)	13.2 ^a^ (11.2–15.8)	14.3 ^a, b^ (11.5–16.2)	15.2 ^b^ (12.6–17.4)	0.011
EFSA	11	below PRI	4.6%	10.3%	1.8%	5.3%	3%	2.5%	0.124
		above PRI	95.4%	89.7%	98.2%	94.7%	97%	97.5%	
Iodine (μg/day)		median (IQ)	92.5 (73.3–116.3)	93.5 (74.8–117.4)	92.3 (75.1–106)	87.1 (69.4–124.6)	94.5 (73.2–115.3)	95.1 (73.5–121.8)	0.870
EFSA	150	below AI	76.6%	76.5%	83.6%	72.4%	80.6%	72.5%	0.459
		above AI	23.4%	23.5%	16.4%	27.6%	19.4%	27.5%	
Zinc (mg/day)		median (IQ)	10.4 (8.7–12.5)	10.5 ^a, c^ (8–12)	9.5 ^a, c^ (8.5–11.8)	9.8 ^a^ (8.2–11.5)	10.8 ^c^ (9.3–12.2)	11.7 ^b^ (9.4–14.7)	0.001
EFSA	14	below PRI	59%	58.8% _a, b_	70.9% _a, b_	72.4% _b_	53.7% _a, c_	42.5% _c_	0.001
		above PRI	41%	41.2% _a, b_	29.1% _a, b_	27.6% _b_	46.3% _a, c_	57.5% _c_	
Magnesium (mg/day)		median (IQ)	290.5 (241.8–345.3)	281 (223.1–354.4)	294.7 (245–336.6)	277 (236.8–321.7)	295 (238–352)	308.4 (251.3–379)	0.205
EFSA	350	below AI	45.1%	47.1%	45.5%	52.6%	44.8%	36.3%	0.357
		above AI	549%	52.9%	54.5%	47.4%	55.2%	63.8%	
Potassium (mg/day)		median (IQ)	2954 (2517.7–3521.4)	2929.8 (2260.1–3399.9)	2951 (2618.7–3520.9)	2919.5 (2491.3–3283.3)	3083 (2411–3695)	3044.4 (2540.5–3716)	0.291
EFSA	3500	below AI	39.6%	42.6%	38.2%	46.1%	32.8%	37.5%	0.547
		above AI	60.4%	57.4%	61.8%	53.9%	67.2%	62.5%	
Phosphorous (mg/day)		median (IQ)	1463.9 (1264.6–1714.5)	1442 ^a, c^ (1202.9–1690.5)	1410.8 ^a^ (1239–1553)	1413.9 ^a^ (1184–1600.3)	1535 ^b, c^ (1339–1788.1)	1581 ^b^ (1303.3–1905.1)	0.003
EFSA	550	above AI	100%	100%	100%	100%	100%	100%	-
Selenium (μg/day)		median (IQ)	112 (91.8–134.2)	118.5 ^a, b^ (89.8–131.8)	103.1 ^a^ (85.6–118.1)	110 ^a, c^ (93.7–131.3)	116 ^b, c^ (91.6–132.2)	116.5 ^b^ (104.2–147)	0.008
EFSA	70	below AI	1.7%	1.5%	5.5%	1.3%	-	1.3%	0.209
		above AI	98.3%	98.5%	94.5%	98.7%	100%	98.8%	
Sodium (mg/day)		median (IQ)	2084.2 (1624.4–2674)	1894 (1600.8–2584.3)	1922 (1541.5–2543)	2067 (1561–2760.4)	2100 (1654–2911)	2361.6 (1852.9–2782)	0.051
EFSA	2000	≤2000 mg/day	45.4%	57.4% _a_	50.9% _a_	47.4% _a_	44.8% _a, b_	30% _b_	0.015
		>2000 mg/day	54.6%	42.6% _a_	49.1% _a_	52.6% _a_	55.2% _a, b_	70% _b_	

Results are expressed as the median (IQ). *p*-values derived through Kruskal–Wallis tests (*p* ≤ 0.05). ^a, b, c^ significant differences between groups (Mann–Whitney U test). _a, b, c_ significant differences between groups (Chi^2^). PRI—Population reference intake. AI—Adequate intake. EFSA—European Food Safety Authority. * Nutritional reference value of calcium according to age.

## Data Availability

The data presented in this study are available on request from the corresponding author due to privacy.
